# Dynamics of antimicrobial resistance in intestinal *Escherichia coli* from children in community settings in South Asia and sub-Saharan Africa

**DOI:** 10.1038/s41564-018-0217-4

**Published:** 2018-08-20

**Authors:** Danielle J. Ingle, Myron M. Levine, Karen L. Kotloff, Kathryn E. Holt, Roy M. Robins-Browne

**Affiliations:** 1 Department of Microbiology and Immunology, Peter Doherty Institute for Infection and Immunity, The University of Melbourne, Melbourne, Victoria Australia; 2 Department of Biochemistry and Molecular Biology, Bio21 Molecular Science and Biotechnology Institute, The University of Melbourne, Melbourne, Victoria Australia; 3 Departments of Pediatrics and Medicine, Center for Vaccine Development, University of Maryland School of Medicine, Baltimore, MD USA; 4 London School of Hygiene and Tropical Medicine, London, UK; 5 Murdoch Children’s Research Institute, Royal Children’s Hospital, Parkville, Victoria Australia; 6 National Centre for Epidemiology and Population Health, Research School of Population Health, The Australian National University, Canberra, Australian Capital Territory Australia; 7 Microbiological Diagnostic Unit Public Health Laboratory, Department of Microbiology and Immunology, Peter Doherty Institute for Infection and Immunity, The University of Melbourne, Melbourne, Victoria Australia

**Keywords:** Bacterial genomics, Bacterial infection, Antibiotics, Antimicrobial resistance

## Abstract

The dynamics of antimicrobial resistance (AMR) in developing countries are poorly understood, especially in community settings, due to a sparsity of data on AMR prevalence and genetics. We used a combination of phenotyping, genomics and antimicrobial usage data to investigate patterns of AMR amongst atypical enteropathogenic *Escherichia coli* (aEPEC) strains isolated from children younger than five years old in seven developing countries (four in sub-Saharan Africa and three in South Asia) over a three-year period. We detected high rates of AMR, with 65% of isolates displaying resistance to three or more drug classes. Whole-genome sequencing revealed a diversity of known genetic mechanisms for AMR that accounted for >95% of phenotypic resistance, with comparable rates amongst aEPEC strains associated with diarrhoea or asymptomatic carriage. Genetic determinants of AMR were associated with the geographic location of isolates, not *E. coli* lineage, and AMR genes were frequently co-located, potentially enabling the acquisition of multi-drug resistance in a single step. Comparison of AMR with antimicrobial usage data showed that the prevalence of resistance to fluoroquinolones and third-generation cephalosporins was correlated with usage, which was higher in South Asia than in Africa. This study provides much-needed insights into the frequency and mechanisms of AMR in intestinal *E. coli* in children living in community settings in developing countries.

## Abstract (web summary)

Genomic analysis of atypical enteropathogenic *Escherichia coli* in South Asia and sub-Saharan Africa uncovers high antibiotic resistance prevalence, which is correlated with antibiotic use.

## Main

Certain pathotypes of *Escherichia coli* are important causes of diarrhoea in children, especially in the developing countries of sub-Saharan Africa and South Asia^1^. Intestinal *E. coli* is also an important source and reservoir of genes that encode antimicrobial resistance (AMR). One pathotype of intestinal *E. coli*, known as atypical enteropathogenic *E. coli* (aEPEC), is defined by the presence of the locus of enterocyte effacement pathogenicity island, and the absence of Shiga toxins (denoting enterohaemorrhagic *E. coli*) and type IV bundle-forming pili (indicating typical EPEC)^2^. Atypical enteropathogenic *E. coli* causes a variety of disease symptoms ranging from sporadic and persistent diarrhoea to asymptomatic carriage^1,3–5^. We recently identified distinct lineages of aEPEC, including ten common clonal groups^6^.

AMR has been reported in *E. coli* from various animal species, the environment and in hospitalized patients globally^7–12^. Many strains exhibit multi-drug resistance (MDR; resistance to one or more agents in at least three different antimicrobial categories^13^). Strains that are resistant to fluoroquinolones and/or produce extended-spectrum β-lactamases (ESBL) or carbapenemases are of particular concern^14^. Although several recent studies of pathogenic *E. coli* from countries in sub-Saharan Africa and South Asia have reported increases in ESBLs^15,16^, as well as increasing resistance to gentamicin^17,18^ and ciprofloxacin^18–22^, these data are mostly derived from *E. coli* responsible for extra-intestinal infections in hospital settings. Thus, there remain major gaps in knowledge of the global prevalence of AMR in human intestinal *E. coli*, particularly in developing nations where the burden of infectious diseases is highest and AMR may result in infections that are unresponsive to treatment^14^.

Enhancing our knowledge of AMR amongst gut-dwelling *E. coli* is important for two reasons: (1) *E. coli* is a leading cause of extra-intestinal infections and strains colonising the gastrointestinal tract of patients are the major reservoir of these infections; and (2) most AMR in *E. coli* is encoded on mobile genetic elements that are transferable between bacteria, thus enabling the rapid dissemination and maintenance of resistance genes between bacteria of different species^23,24^. Although antimicrobials are not recommended for the treatment of uncomplicated gastroenteritis, they are commonly administered to diarrhoeic children in developing countries to treat dysentery^25^ and prolonged diarrhoea, of which aEPEC is a major cause^4,5^.

Here we present AMR data for 185 aEPEC isolates collected during the Global Enteric Multicenter Study (GEMS)^1,26^. Using phenotypic susceptibility data and whole-genome sequence analysis, we determined the prevalence, mechanisms of resistance and potential drivers of variation in AMR profiles. These isolates, collected from healthy children living in a community setting and children with diarrhoea at seven sites in sub-Saharan Africa and South Asia, provided a unique opportunity to investigate the prevalence of AMR in intestinal bacteria that were not selected on the basis of AMR profile.

## Results

### Antimicrobial susceptibility profiles

**Fig. 1:**
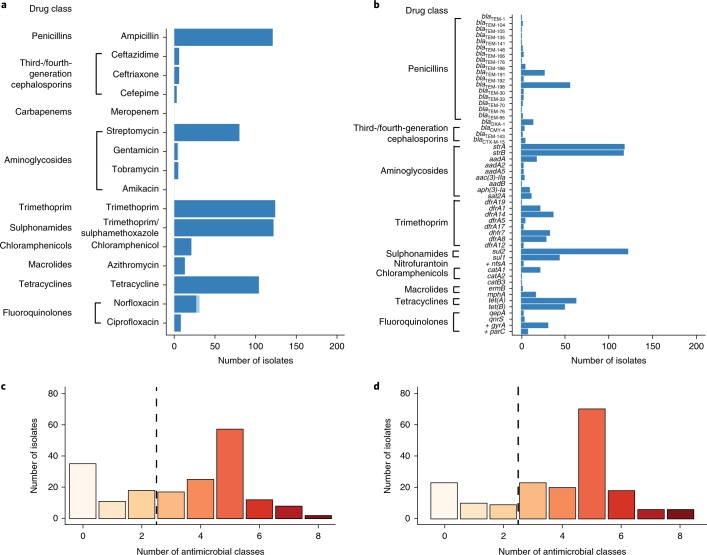
Prevalence of AMR-associated gene content and AMR phenotypes in 185 aEPEC isolates. **a**,**c** AMR phenotypes of the aEPEC isolates. **a**, AMR profiles grouped by the drug class to which aEPEC strains were phenotypically resistant. **c**, Histogram illustrating the number of drug classes to which aEPEC strains were phenotypically resistant. **b**,**d** AMR-associated gene content of aEPEC strains. **b**, Genes detected in the genomes associated with AMR are shown to the left of the graph and are grouped by drug class. Gene that contain point mutations that result in AMR and that are not acquired through horizontal gene transfer are indicated with a cross. **d**, Histogram illustrating the number of classes to which aEPEC strains were detected as having AMR-associated gene content.

Susceptibility testing of 185 aEPEC isolates (Supplementary Table 1) to 16 antimicrobials (Supplementary Table 2) revealed resistance to 14 of the drugs investigated (Fig. 1a; Supplementary Table 3), with 121 MDR isolates (65%; Fig. 1c). No resistance was detected to the ‘last-line’ drugs, amikacin (an aminoglycoside) or meropenem (a carbapenem). Only 35 isolates (19%) were susceptible to all drugs tested: 17 from cases (18%) and 18 from controls (20%). Resistance to ‘older’ antimicrobials was common, with 121 (65%) isolates resistant to ampicillin, 124 (67%) to trimethoprim, 122 (66%) to trimethoprim/sulphamethoxazole and 104 (56%) to tetracycline (Fig. 1a). Approximately half (*n* = 96, 52%) the isolates were resistant to three or more of these drugs. Streptomycin resistance was common (43%), although this antibiotic is not used to treat diarrhoea. Resistance to other aminoglycosides tested was rare (3%), with five isolates from India resistant to tobramycin, four of which were also resistant to gentamicin. Fluoroquinolone resistance was relatively infrequent, with 31 isolates (17%) resistant to norfloxacin and 8 (4%) resistant to both norfloxacin and ciprofloxacin. Resistance to chloramphenicol (11%) and azithromycin (7%) was also infrequent. Among the β-lactam antibiotics, ampicillin resistance was common (65%), but resistance to ceftriaxone (3%), ceftazidime (3%) and cefepime (2%) was rare (Fig. 1a,b).

### Genetic determinants of AMR

The genomes of the 185 aEPEC isolates were screened for known genetic determinants of AMR, including horizontally acquired genes and point mutations in chromosomal genes associated with resistance to fluoroquinolones and nitrofurantoin (Fig. 1b; Supplementary Fig. 1 and Supplementary Table 4). More than forty different acquired AMR genes were detected, along with four point mutations (two in *gyrA*, one in *parC* (both *gyrA* and *parC* are associated with quinolone resistance) and one in *nfsA* (associated with resistance to nitrofurantoin)). Extensive diversity of AMR genotypes was observed, with 104 distinct combinations of AMR determinants across the 185 isolates (Fig. 1b, Supplementary Fig. 1). Nevertheless, four acquired AMR genes were detected in more than half the isolates. These were alleles of *bla*_TEM_ (ampicillin), *strA* and *strB* (streptomycin), and *sul2* (sulphonamides). Alleles of dihydrofolate reductase (*dfr*) genes encoding trimethoprim resistance were detected in 132 (71%) isolates. The most common of these were *dfrA1* (12%), *dfrA5* (23%), *dfrA7* (20%) and *dfrA8* (16%).

### Investigation of mobile genetic elements associated with transfer of AMR genes

**Fig. 2:**
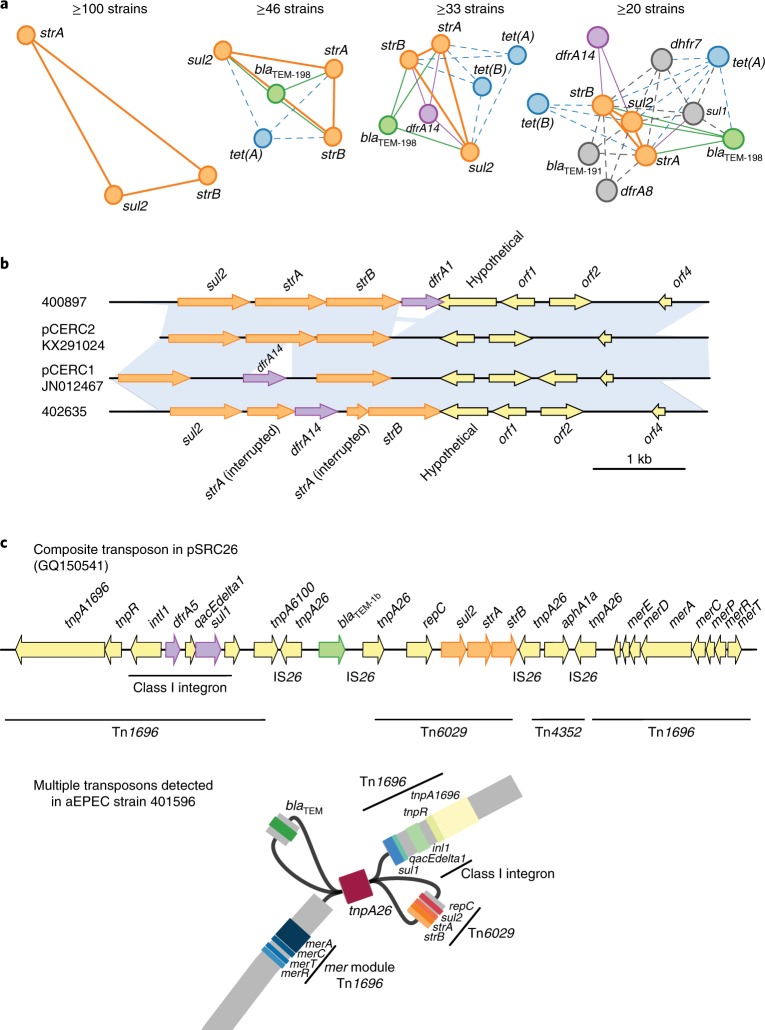
Co-occurrence and characterization of common mobile elements of AMR-associated genes in aEPEC strains. **a**, Visualization of AMR gene co-occurrence networks among GEMS aEPEC strains, using different frequency thresholds. Solid lines join genes that occur together on known mobile elements at a high frequency; dashed lines join genes that occur together on known mobile elements at a lower frequency. The mean frequency of co-occurrence in strains was 6.4, with standard deviation 13.1. **b**, BLAST comparison of pCERC1 and pCERC2 to representative plasmids from two GEMS aEPEC strains (400897 and 402635). Blue shading indicates regions of sequence homology. **c**, Gene arrangement in composite transposons previously identified in *Salmonella* plasmid pSRC26 and assembly graph for GEMS aEPEC strain 401596, showing how this composite transposon appears in assemblies inferred from short-read Illumina data. The assembly graph was visualized in Bandage; coloured blocks indicate BLAST hits to AMR genes as labelled.

As MDR was common in the bacteria studied (Fig. 1), we hypothesized that this was due to the co-transfer of groups of AMR genes via mobile elements. The pairwise co-occurrence matrix of AMR genes was sparse (Supplementary Fig. 2), with only a few clusters of genes frequently detected together in the same genome. The mean co-occurrence value across all gene pairs was 6.4 strains. Figure 2a shows AMR gene co-occurrence networks, constructed using different thresholds for co-occurrence across the aEPEC collection. The most common gene network comprised *sul2*, *strA* and *strB*, which co-occurred in 112 genomes (61%); the combination of *sul2*, *strA* and *strB* occurred with *bla*_TEM-198_ in 46 (25%) and with *bla*_TEM-191_ in 20 (11%; and Supplementary Fig. 2 and Fig. 2a) genomes. Using a minimum threshold of co-occurrence in ≥20 strains (mean plus s. d. of all co-occurrence values), we detected a large network of genes comprising *sul2*, *strA*, *strB* and *bla*_TEM-198_, as well as *sul1*, *bla*_TEM-191_, *dfrA14*, *dfrA8*, *dfrA7*, *tet(A)* and *tet(B)*.

Resolving the genetic context of AMR genes is generally not possible using short-read sequence data, because repeated sequences (such as insertion sequences) and variable plasmid copy numbers cause uncertainty in the de novo assembly graphs^27,28^. We therefore sought only to make broad classifications about the potential mobile elements associated with the AMR gene networks present in the aEPEC genomes, through comparison with elements that have previously been found to mobilize these combinations of genes.

The genes *sul2*, *strA* and *strB* frequently move together on small (approximately 6,000 base pairs (kbp)) plasmids related to pCERC2^29^ (Fig. 2b). The combination of the pCERC2 plasmid backbone and the *sul2*, *strA* and *strB* sequences was present in 42 isolates, 40 of which (95%) also carried *dfrA* gene sequences, including *dfrA1*, *dfrA14* and *dfrA8*. In some genomes, the plasmid sequences could be resolved completely, showing that the *dfr* gene was located on the plasmid. For example, GEMS strain 400897 carried *dfrA1* gene adjacent to *strB* on a pCERC2-like plasmid, while GEMS strain 402635 carried *dfrA14* inserted within *strA* as in pCERC1^29^ (Fig. 2b).

The *sul2*, *strA* and *strB* genes also occur together with *bla*_TEM_ genes (predominately *bla*_TEM-198_) in transposon Tn*6029*, which is commonly found in *E. coli* in a range of distinct plasmid backbones^27,29,30^ (Fig. 2c). Tn*6029* is mobilized by the flanking copies of IS*26*. A third copy of IS*26* is located between *bla*_TEM_ and the other AMR genes, resulting in separation of the transposon into two separate contigs in short-read assemblies (Fig. 2c). We detected the presence of both Tn*6029* contigs in 33 genomes, which are therefore likely to carry the complete transposon. As the flanking IS*26* sequence is present in many different locations, we could not determine the insertion site of Tn*6029* within the draft genomes. However, Tn*6029* is frequently located within Tn*1696*, which includes a class I integron that carries variable AMR genes (often including *dfr* genes) within the cassette, and *sul1* downstream of the cassette. For an example of a composite transposon structure from *Salmonella* plasmid pSRC26^31^, see Fig. 2c. In total, we identified class I integron sequences in 38 aEPEC genomes, 26 of which included both Tn*6029* contigs. An example assembly graph, showing how these contigs are connected to one another in a manner consistent with previously sequenced composite transposons, is shown in Fig. 2c. Overall, we identified five different integron gene cassettes, the most prevalent of which carried *dfrA7* (*n* = 30 genomes, including 25 with Tn*6029*), whereas the others carried *dfrA1* (*n* = 4), *dfrA1* and *aadA* (*n* = 1 genome, which also carried Tn*6029*), *dfrA17* and *aad5* (*n* = 2), and *dfrA5* (*n* = 1).

We found two common genes that encode tetracycline resistance efflux pumps. The most prevalent was *tet(A)*, which was found in 63 (34%) aEPEC genomes. Although *tet(A)* is associated with the Tn*1721* transposon, the full transposon was detected in only three genomes. The *tet(B)* gene was detected in 50 (27%) genomes, five of which also carried *tet(A)*. The *tet(B)* gene can be mobilized by Tn*10*, which is flanked by IS*10* genes, but the complete transposon was present in only two genomes. The linkage of *tet(A)* or *tet(B)* to other AMR-related elements was not resolvable from the draft genome assemblies, however both were found in association with other common AMR genes (Fig. 2a).

Although it is not possible to resolve plasmid sequences from draft short-read assemblies or determine linkage between specific plasmid replicons and AMR genes^32^, our screening for markers of plasmid replicons revealed several that are often associated with large AMR plasmids (Supplementary Fig. 1d). The most prevalent amongst the aEPEC collection were FII (*n* = 131, 71%) and FIBA (*n* = 104, 56%). Notably, F plasmids are also associated with the carriage of genes for virulence determinants, such as adhesins of enteropathogenic *E. coli*, but our data did not permit the determination of which plasmids were associated with AMR genes versus virulence genes. An IncC plasmid replicon (also known as IncA/C) was detected in four genomes that carried *bla*_TEM-198_ but not *sul2, strA* or *strB*. It was not possible, however, to determine whether the *bla*_TEM-198_ gene was located on this plasmid.

### Prediction of AMR phenotypes from genotypes

In vitro resistance was largely explained by the presence of known genetic determinants of AMR (Fig. 1, Table 1). For most drugs, the detection of resistance genes was both sensitive (>95%) and specific (>90%) in predicting AMR phenotype. The frequency of very major errors (failure to detect phenotypic resistance) exceeded the minimum acceptable threshold of 1.5% for five drugs (Table 1). These were: ampicillin (4.9%), streptomycin (2.2%), trimethoprim (2.2%), trimethoprim/sulphamethoxazole (2.2%) and tetracycline (2.2%). Major errors (predicting resistance when none is present) were also detected for these and several other antimicrobials (Table 1). The highest major error rates were observed for streptomycin (26.5%), ampicillin (7.0%), trimethoprim (5.9%), trimethoprim/sulphamethoxazole (5.4%) and tetracycline (4.3%). The sensitivity, specificity, positive predictive value (PPV) and negative predictive value (NPV) for each drug are shown in Supplementary Table 5. Sensitivity and NPV were greater than 90% for all drugs tested with the exception of azithromycin (85% sensitivity) and ampicillin (85% NPV). Specificity and PPV were more variable, reflecting the error rates summarized above (Table 1).

**Table 1:** Comparison of phenotypic and genotypic AMR profiles of 185 aEPEC isolates

	Resistant phenotype	Resistant genotype	Very major error^a^	Susceptible phenotype	Susceptible genotype	Major error^b^
**β-lactams**						
Ampicillin	121	112	**9 (4.9%)**	64	51	**13 (7.0%)**
Cefepime	3	3	0	182	178	4 (2.2%)
Ceftriaxone	6	6	0	179	174	5 (2.7%)
Ceftazidime	6	6	0	179	174	5 (2.7%)
Meropenem	0	0	0	185	185	0
**Aminoglycosides**						
Streptomycin	80	76	**4 (2.2%)**	105	56	**49 (26.5%)**
Gentamicin	5	5	0	180	180	0
Tobramycin	5	5	0	180	180	0
Amikacin	0	0	0	185	185	0
**Folate pathway inhibitors**						
Trimethoprim	124	120	**4 (2.2%)**	61	50	**11 (5.9%)**
Trimethoprim/sulphamethoxazole^c^	122	118	**4 (2.2%)**	63	53	**10 (5.4%)**
**Nitrofurantoin**						
Nitrofurantoin	0	0	0	185	182	3 (1.6%)
**Chloramphenicol**						
Chloramphenicol	21	19	2 (1.1%)	164	160	4 (2.2%)
**Macrolides**						
Azithromycin	13	11	2 (1.1%)	172	166	**6 (3.2%)**
**Tetracyclines**						
Tetracycline	104	100	**4 (2.2%)**	81	73	**8 (4.3%)**
**Fluoroquinolones**						
Ciprofloxacin^d^	8	8	0	177	173	4 (2.2%)
Norfloxacin	31	30	1 (0.5%)	154	149	5 (2.7%)

^a^Very major errors (resistant isolate genotyped as susceptible) at frequencies >1.5% are shown in bold.

^b^Major errors (susceptible isolate genotyped as resistant) at frequencies >3% are shown in bold.

^c^When two genes are required for resistance, both were required for genotypic resistance: *strA* and *strB* for streptomycin, and a *dfr* gene plus a *sul* gene for resistance to trimethoprim/sulphamethoxazole.

^d^At least one point mutation in *gyrA* and a second in *gyrA*, *gyrB*, *parC* or *parE.*

### Potential sources of variation in AMR profiles

**Fig. 3:**
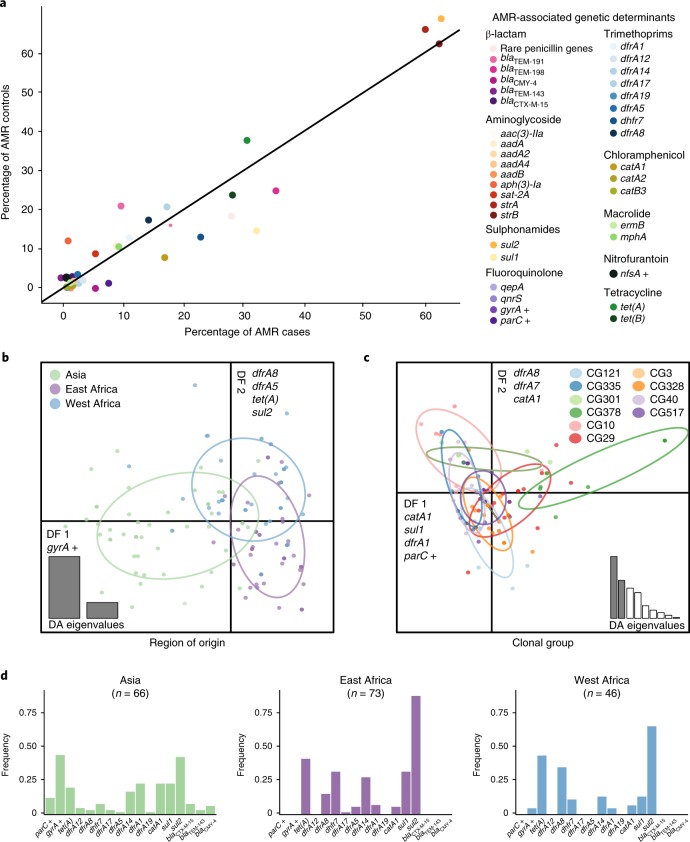
AMR gene content is explained by region of isolation, not disease status or clonal group. **a**, Frequencies of AMR-associated genes in aEPEC by case or control status. Genes encoding AMR are shown to the right of the graph and are grouped by drug class. **b**,**c**, Discriminant analysis of principal components based on known genetic determinants of AMR. The graphs display the discriminant functions (DF) that best discriminate isolates into region of isolation (**b**, *n* = 185) or clonal group (**c**, *n* = 137). Data points are coloured coded according to their demographic group (see legend) and the genetic determinants most correlated with the DFs are labelled on the DF axes. Eigenvalues, corresponding to the ratio of the variance between groups over the variance within groups for each principal component in the discriminant function, are displayed in the insets. **d**, Frequency of AMR genetic determinants that differed between Asia, East Africa and West Africa. Genes that contain point mutations that result in AMR and that are not acquired through horizontal gene transfer are indicated with a cross.

Given the diversity of AMR profiles in our aEPEC strains, we determined whether the distribution of AMR genes was associated with disease status, phylogenetic lineage or the geographic location from which each strain was isolated. First, we compared the frequency of AMR phenotypes and genotypes among aEPEC isolated from diarrhoea cases and asymptomatic controls. Only data from confirmed cases (*n* = 94) and controls (*n* = 88) were used for this analysis. For each drug class, neither AMR phenotype nor AMR predicted from genotype, were statistically different between cases and controls (Supplementary Table 6). The frequencies of individual AMR genes were also similar in isolates obtained from cases and from controls (Fig. 3a). As AMR determinants were equally distributed among cases and controls, all isolates were pooled for further analysis of lineage and region.

Discriminant analysis of principal components^33^ on the binary matrix of AMR genetic determinants (Supplementary Table 4; Fig. 3b,c) revealed that the first 20 principal components accounted for >93% of variation in AMR profiles and were retained for discriminant analysis by phylogenetic lineage (clonal groups as defined previously^6^; Supplementary Fig. 1) or by geographic origin (East Africa, West Africa and Asia; Fig. 3 and Supplementary Fig. 3).

**Fig. 4:**
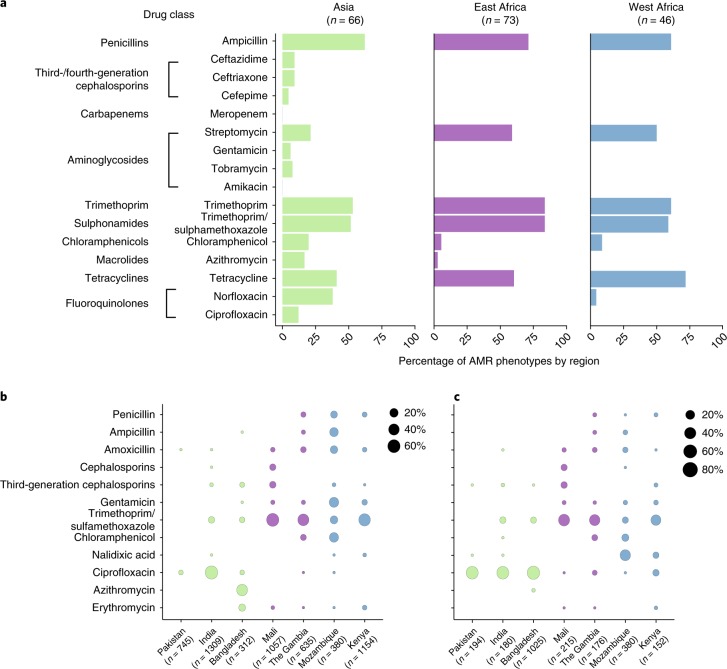
AMR phenotypes by region and antimicrobial use at study sites. **a**, AMR phenotypes of GEMS isolates, stratified by region of isolation. **b**,**c**, Percentage of antimicrobials prescribed to patients with watery diarrhoea (**b**) or dysentery (**c**) at each of the seven study sites.

Variation in AMR was not associated with clonal group, apart from CG378 which was characterized by the absence of the most common AMR genes: *bla*_TEM_ variants, *sul2*, *tet(A*) and *tet(B)*, and the presence of the uncommon *catA* gene, detected in 7 of 9 CG378 isolates compared to 15 of 176 non-CG378 (*P* < 10^-4^, Fisher’s exact test, two-tailed). Variation in AMR determinants was associated with the region of origin (Fig. 3b and Supplementary Fig. 3b). Discriminant function 1 (DF1) separated Asian from African isolates and was associated with *gyrA* single nucleotide polymorphisms; DF2 separated East from West African isolates and was associated with *dfrA8*, *dfrA5*, *tet(A)* and *sul2*. Figure 3d shows the distribution of the *dfrA* alleles across the GEMS sites. For example, *dfrA1* predominated at Asian sites and *dfrA8* was most common at West African sites, whereas *dfrA14* and *dhfr7* were common in Mozambique and Kenya, respectively. Further, *tet(A)* was more common at West and East African sites than in Asia. These genetic differences were reflected in AMR phenotypes, as resistance to ciprofloxacin (*n* = 8, 12%) and third-generation cephalosporins (ceftazidime and ceftriaxone, both *n* = 6; 9%) was identified only in strains from Asia, while resistance to tetracycline was more common in African (East Africa, *n* = 44, 60%; West Africa, *n* = 33, 72%) than in Asian isolates, *n* = 27, 41 %; *P* < 0.05, Fisher’s exact test, two-tailed; Fig. 4a).

### Differences in local antimicrobial drug usage

**Fig. 5:**
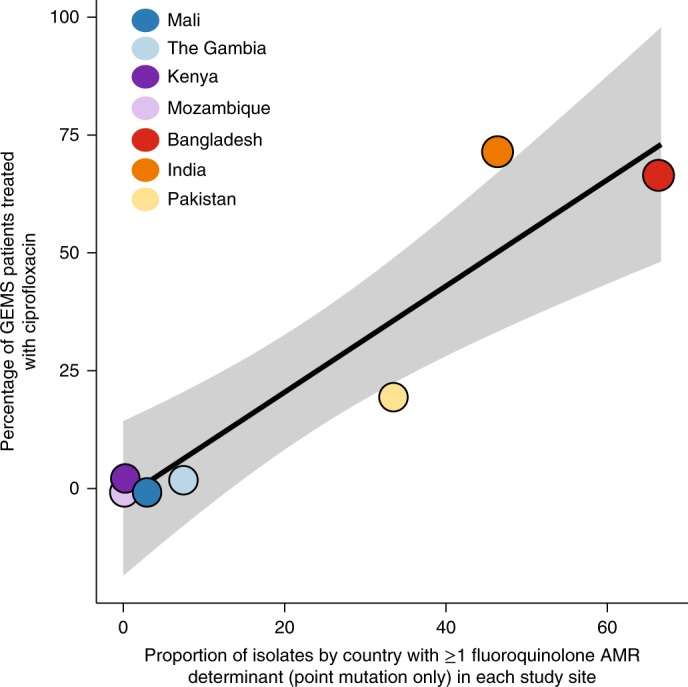
Relationship between the use of ciprofloxacin at GEMS study sites and ciprofloxacin resistance in aEPEC. Linear regression (mean ± 95% confidence interval shaded in grey) of the proportion of aEPEC containing genetic determinants of ciprofloxacin resistance (from point mutations only) versus the proportion of watery diarrhoea and dysentery cases treated with ciprofloxacin at GEMS study sites.

The broad patterns of antimicrobial use in the treatment of diarrhoea across the GEMS study sites showed that trimethoprim/sulphamethoxazole and penicillins were used more frequently at African sites, whereas macrolides (azithromycin) and fluoroquinolones (in particular ciprofloxacin) were used more frequently in Asia (Fig. 4b,c). These patterns of antimicrobial use showed some association with AMR phenotypes, insofar as we observed higher levels of both usage and resistance for azithromycin and fluoroquinolones at Asian sites, and to trimethoprim at East African sites (Fig. 4). We could not formally test these associations, however, due to the small numbers of observations at some study sites and minor variations in usage of most drugs between the sites. We therefore investigated the associations between usage and resistance for the two drugs that showed substantial usage (>10%) at three or more study sites: ciprofloxacin and trimethoprim. Across the seven sites, ciprofloxacin usage was significantly associated with the prevalence of substitutions in the quinolone resistance-determining regions (QRDRs), *gyrA* and *parC* (Coefficient of determination (*R*^2^) = 0.87, *P* *=* 0.002; Fig. 5). By contrast, trimethoprim usage was not associated with the prevalence of horizontally acquired *dfr* genes that confer resistance to the drug (*R*^2^ = 0.04, *P* > 0.5).

## Discussion

AMR and usage data reported here were collected at seven study sites in Asia and Africa, using the same protocols thus enabling comparisons between the sites^26,34^. No national AMR surveillance data are available from these countries; and AMR data on *E. coli* in these countries pertain mostly to isolates causing extra-intestinal infections, and to a limited number of drugs (mainly third-generation cephalosporins and fluoroquinolones)^14,19,21,22,35^. Furthermore, publicly available background data on antimicrobial usage at the seven study sites are limited. For example, the IMS Health MIDAS database includes usage data for India, Pakistan and Bangladesh reported in aggregate without detailed methods of data collection or interpretation; and no data for Mozambique, The Gambia, Kenya and Mali.

Nearly half of all isolates were resistant to penicillins, trimethoprim and tetracyclines. The rates of resistance to these drugs were generally lower amongst Asian isolates (21–62%) than in Africa isolates (59–84%); whereas resistance to newer drugs such as ceftriaxone, fluoroquinolones and azithromycin were detected at the Asian sites (Fig. 4a). These patterns were broadly consistent with the antimicrobial usage data at the corresponding study sites, which showed that ciprofloxacin and azithromycin were commonly used to treat diarrhoea in Asia, whereas trimethoprim-sulphamethoxazole was the mainstay of treatment in Africa (Fig. 4b,c). The high frequencies of ciprofloxacin resistance at the Asian sites are similar to rates previously reported among clinical cases of intestinal *E. coli* (including multiple pathotypes) in these countries^17,19,21,22,36^. We detected ESBL-producing isolates at the Asian, but not the African, study sites. Much higher levels have been reported from extra-intestinal *E. coli* infections (including bacteraemia) in hospitals in Asia and Africa^16,19,35^, we speculate that this may reflect selection due to use of third-generation cephalosporin at higher rates in hospitals than in the community, and/or the dissemination of the ESBL-producing extra-intestinal *E. coli* lineages, such as ST131.

In agreement with our data relating to AMR phenotypes, we found that the genetic determinants of resistance were similar in bacteria isolated from diarrhoeal cases and asymptomatic controls (Fig. 3a, Supplementary Table 6) and were not associated with the clonal lineage of the strain, but associated with the geographic region where the bacteria were isolated (Fig. 3b,c; Supplementary Fig. 3). These findings are consistent with the hypothesis that differences in the frequencies of AMR-encoding genes in different regions reflect selection due to differences in antimicrobial exposure (Fig. 4b,c). In strong support of this explanation, mutations in the QRDRs of *gyrA* and *parC* were significantly associated with the frequency of ciprofloxacin use across the seven study sites (Fig. 5). The same pattern of point mutations in the QRDRs of chromosomal genes has been observed in other Enterobacteriaceae associated with South Asia, including *Salmonella enterica* serovar Typhi^37,38^ and *Shigella sonnei*^39^.

The situation was more complex for horizontally transferred AMR genes associated with resistance to older drugs. Although individual *dfr* alleles were distributed differently across sites (Fig. 3d) and contributed to orthogonal components of the regional discriminant function (Fig. 3b), the overall prevalence of *dfr* genes was relatively high (50–90%) at each site and not significantly associated with use of trimethoprim for diarrhoea (Figs. 3d and 4b,c). Similarly, genes encoding resistance to ampicillin, streptomycin and tetracycline were common at sites where these drugs were seldom or never used for the treatment of diarrhoea. We also found evidence of several common elements mediating AMR to these older drugs, including small plasmids and class I integrons (Fig. 2). This could be due to: (1) a lack of fitness cost associated with these resistances, resulting in maintenance of the genes in the *E. coli* population after drug usage declines^8,10,30,40,41^; (2) co-selection for resistance to multiple drugs whose associated genes are present on the same mobile elements^42,43^; and/or (3) selection due to drug exposure unrelated to the treatment of diarrhoea. We could not distinguish between hypotheses using our data, although exposure to antimicrobials from other sources is quite likely. For example, although we found that trimethoprim, alone or combined with sulphamethoxazole, was used less frequently in India than alternative agents for the treatment of diarrhoea, other studies have reported frequent use of trimethoprim in hospitals, community settings^36^ and agriculture in India; there is also evidence of its presence in the environment, particularly surface waters^44^. Future studies would benefit from additional data on clinical and agricultural antimicrobial exposures, and assays for selected antimicrobials agents in urine and the environment.

We were also unable to determine the precise location of most AMR genes and associated mobile elements from our short-read sequence data. Further experiments such as conjugation or long-read sequencing^38,45,46^ could resolve this in future. It is notable, however, that the pCERC-like plasmids (which often carried resistance to streptomycin, sulphonamides and trimethoprim) are common in *E. coli*, possibly because their small size (~6 kb) imposes a low fitness cost^29,47^. It is also notable that these and many of the other AMR genes we detected are also associated with composite transposons that can integrate into the bacterial chromosome, where they are maintained at lower fitness cost than large resistance-encoding plasmids^27,31,38^. Many of the mobile elements we detected have been reported widely in *E. coli* and other Enterobacteriaceae from human intestinal and extra-intestinal samples and animal samples^29,38,48–50^. Atypical enteropathogenic *E. coli* has multiple reservoirs and our collection included extensive phylogenetic diversity within the *E. coli* population (90 unique lineages^6^); and we found no differences in AMR between isolates cultured from cases and asymptomatic colonization. Thus, although the isolates were not collected for the specific purpose of AMR surveillance, they may be broadly representative of intestinal *E. coli* in these settings.

Our data fill important information gaps concerning the prevalence of AMR among intestinal *E. coli* in children from developing countries in Africa and Asia. In particular, our study showed that resistance to multiple ‘older’ drugs (ampicillin, tetracycline, streptomycin and trimethoprim-sulphamethoxazole) was common at all sites, and that resistance to ‘newer’ antimicrobials, such as fluoroquinolones and azithromycin, has emerged only in Asian sites where these drugs are used in the management of diarrhoea. Resistance to older drugs was also common at these sites, such that only Asian isolates were resistant to seven or eight categories of antimicrobials, indicating that changing patterns of antimicrobial use leads to an accumulation of resistance determinants rather than their replacement.

## Methods

### aEPEC isolates and corresponding whole-genome sequences

A total of 185 confirmed aEPEC isolates from children aged 0–5 years at GEMS sites located in The Gambia, Mali, Kenya, Mozambique, Bangladesh, India and Pakistan^34^ were included in the analysis^1,6,26,34^. Their collection, selection for sequencing, whole-genome sequencing and phylogenomic analysis have been described previously^6^. Briefly, the isolates were mostly from fecal samples in which aEPEC alone (or with *Giardia lamblia*) was the only pathogen detected, where a pure culture could be obtained. All such isolates from diarrhoeal cases were sequenced (*n* = 94); controls matched for age, sex and study site were also included (*n* = 88). Three isolates were from children whose case/control status was uncertain. Faecal samples were collected at the study sites before antimicrobial treatment, although previous exposure to antimicrobials from other sources cannot be ruled out. Control children were also not receiving any antimicrobial treatment.

Whole-genome sequences were generated for all 185 aEPEC isolates at the Wellcome Trust Sanger Institute using the Illumina HiSeq platform (100 bp paired-end reads) and assembled using Velvet, as described previously^6,51^. Details of the individual isolates, accession numbers for the corresponding genome sequence reads and assemblies (deposited collectively under BioProject ERP001141), and associated metadata are provided in Supplementary Table 1.

### Phenotypic characterization of AMR profiles

Antimicrobial susceptibility testing to 16 antimicrobials was performed using the VITEK2 (bioMérieux) system or an agar-dilution method. A summary of the drugs, testing methods, and the minimum inhibitory concentration (MIC) breakpoints used to determine susceptible, intermediate or resistant status for each drug is shown in Supplementary Table 2. The controls used were three reference *S. enterica* isolates with known resistance profiles that were kindly provided by the Microbiological Diagnostic Unit Public Health Laboratory. These strains had the following profiles: (1) susceptible to all drugs tested; (2) resistant to ampicillin, streptomycin, tetracycline, chloramphenicol, sulphathiazole, trimethoprim, kanamycin, spectinomycin and gentamicin; and (3) resistant to streptomycin_mod_, tetracycline, kanamycin, nalidixic acid and ciprofloxacin.

For VITEK2 assays, pure isolates were streaked on MacConkey agar plates and incubated at 37 °C overnight. Isolates were then subcultured onto horse blood agar (HBA) plates for fresh culture and incubated overnight at 37 °C. One to three colonies were selected from each HBA plate and suspended in saline to an absorbancy of ~0.5 MacFarlane Units before being subjected to VITEK2 analysis. The raw MIC data from the VITEK2 assays are shown in Supplementary Table 7.

Susceptibility to streptomycin, chloramphenicol, azithromycin and tetracycline were determined using an agar-dilution method. Bacterial suspensions were prepared as described above. To each of 32 stainless steel wells, 450 µl nutrient broth containing 0.05% agar was added, followed by 50 µl bacterial suspension. Each Mueller Hinton agar antimicrobial-containing plate for susceptibility testing and two control Mueller Hinton and MacConkey agar plates were inoculated using a 32-pin replicator. Each pin delivered 2 µl to the plate such that the final number of colony-forming units in each sample was ~10^4^. Plates were incubated overnight at 37 °C and inspected the next day. Growth on an antimicrobial-containing Mueller Hinton plate was recorded as phenotypically resistant to the drug, whereas no growth was recorded as susceptible.

The European Committee on Antimicrobial Susceptibility Testing (EUCAST) MIC breakpoints (version 6) were used where available^52^. Differences exist between the EUCAST and Clinical and Laboratory Standards Institute (CLSI) guidelines in terms of MIC breakpoints and the drugs to be tested. As tetracycline does not have defined MIC breakpoints under the EUCAST scheme, we used the CLSI MIC breakpoint. Streptomycin and azithromycin do not have established MIC breakpoints under either scheme^53,54^. Previous research proposed a breakpoint of 16 µg ml^-1^ for streptomycin in *E. coli*^54^. Little information is available on the MIC distribution of azithromycin for *E. coli*. A breakpoint of 16 µg ml^-1^ has been proposed for *S. enterica* based on a study in which the majority of isolates displayed MICs of 4–8 µg ml^-1^(ref. ^53^). For the present study, the breakpoint MIC for each of these drugs was set at the conservative value of 16 µg ml^-1^. The antimicrobial susceptibility data for each isolate are shown in Supplementary Table 3.

### Detection of AMR genes

An SRST2-formatted version of the ARG-ANNOT AMR gene database^55^ was downloaded from https://github.com/katholt/srst2. All sequence read sets were screened against the database using SRST2 to detect the presence of known acquired resistance genes in each genome^56^. The β-lactamase genes, *ampC1*, *ampC2* and *ampH*, were excluded from analysis, as in *E. coli* they are core genes that normally do not confer antibiotic resistance. The results were transformed into a binary table in R to indicate presence/absence of acquired resistance gene alleles (Supplementary Table 4).

### Detection of single nucleotide polymorphisms conferring resistance to fluoroquinolones and nitrofurantoin

Chromosomal mutations, known to be associated with resistance to fluoroquinolones in *E. coli*, were extracted from the genome-wide single nucleotide polymorphism calls obtained previously based on mapping the reads to the *E. coli* strain 12009 O103:H2 reference genome^6^. These included specific mutations in the quinolone resistance-determining regions of *gyrA*, *gyrB*, *parC* and *parE*^57^, and non-synonymous substitutions in *nfsA* (residues 11–15) that confer resistance to nitrofurantoin^58,59^.

### Statistical analysis of AMR phenotype prediction from genotype

All statistical analyses were performed using the R Stats Package version 3.4.0. The ability of genotypes to predict drug susceptibility phenotypes was assessed by comparing antimicrobial susceptibility phenotypes (S, I and R) with the presence of known AMR-associated genes and mutations. Errors in predicting antimicrobial susceptibility were characterized as very major (calling a resistant isolate susceptible) or major (calling a susceptible isolate resistant)^60,61^. The currently accepted standards for very major error and major error rates are <1.5% and <3%, respectively^60^. Here, very major errors were said to have occurred when an isolate was phenotypically resistant, but no known resistance genes or mutations were detected, while major errors were made when an isolate carried known resistance determinant(s) but was phenotypically susceptible. Statistical analysis to determine sensitivity, specificity, PPV and NPV were calculated in the epiR package (v0.9-93)^62^ for R, using the *epi.stats* function with 0.95 confidence intervals for each antimicrobial tested.

### Genomic reconstruction of demographic groups by discriminant analysis of principal components

The matrix of AMR genetic determinants (specifically the data in Supplementary Table 4 with only AMR genetic determinants used and isolates with two point mutations in *gyrA* treated as present to generate a binary matrix) was used as the input for discriminant analysis of principal components that was implemented in the adegenet package (v2.1.0) in R^33^. The first 20 principal components, which together explained >93% of the variance in AMR gene content, were retained for discriminant analysis to explore the ability of principal components to discriminate between groups of strains defined by geographic region of origin (West Africa: The Gambia and Mali; East Africa: Kenya and Mozambique; and Asia: Bangladesh, India and Pakistan) or clonal group membership. The two principal components contributing the most to discriminant analysis were plotted and labelled with the genetic determinants whose variation contributed the most to those components. The posterior group membership probabilities for each discriminant function were also plotted.

### Construction of co-occurrence network

A pairwise co-occurrence matrix of acquired AMR genes was constructed by transforming the binary AMR gene content matrix in R. The co-occurrence relationships were visualised between all pairs of genes using the pheatmap package (v1.0.8) in R (https://CRAN.R-project.org/package=pheatmap) (Supplementary Fig. 2). Networks of co-occurring genes, in which nodes represent genes and edges represent a frequency of co-occurrence exceeding a given threshold (set to ≥20, ≥33, ≥46, ≥100 genomes), were visualized in R using the igraph package (v1.1.2)^63^ in R.

### Plasmid replicon screening

An SRST2-formatted version of the PlasmidFinder database^64^ was downloaded from https://github.com/katholt/srst2 for the detection of 80 known plasmid replicon marker sequences. All sequence read sets were screened against the database using SRST2 to detect the presence of these replicons in each genome. The results were transformed into a binary table in R to indicate presence/absence.

### Visualization of AMR and plasmid genotypes against a core gene tree

A subtree representing the relationships between the 185 GEMS aEPEC isolates was extracted from the full core phylogeny we published previously^6^ by pruning all other tips using R packages ape (v5.1)^65^ and geiger (v2.0.6)^66^. The presence of acquired AMR genes, mutations and plasmid replicons was plotted as a heatmap against the phylogeny using the plotTree function for R (https://github.com/katholt/plotTree).

### Investigation of mechanisms of AMR gene mobilisation

Common AMR-associated genes that were shown to co-occur, specifically *bla*_TEM-198_, *sul1*, *sul2*, *strA*, *strB*, multiple *dfrA* alleles, *tet(A)* and *tet(B)*, were further investigated in the aEPEC genome assemblies to determine whether they were carried on the same mobile elements. The aEPEC genome assemblies generated previously^6^ were interrogated with BLAST (v2.3.30), using as queries the AMR genes and the sequences of the plasmids pCERC1 (accession JN012467) and pCERC2 (accession KX291024), and the transposons Tn*6029* (accession GQ150541)^27^, Tn*1721* (accession X61367)^67^ and Tn*10* (accession AF223162)^68^. For example, if the pCERC2 backbone and AMR genes were all detected on a single contig in the genome assembly, we inferred that these genes were moving together on a pCERC2-related plasmid. Two representative aEPEC isolates that were identified as harbouring a pCERC2-like plasmid backbone with different *dfr* gene insertions (strains 402635 and 400879) were selected as representatives for further analysis. These genomes were re-assembled with Unicycler (v0.2.0)^69^, annotated using Prokka (v1.12)^70^ and compared to the reference sequences for pCERC1 and pCERC2 using BLAST. The comparisons were then explored using Artemis Comparison Tool^71^ and plotted with genoplotR (v0.8.7) in R^72^.

Atypical enteropathogenic *E. coli* isolates were inferred to be likely carriers of Tn*6029* or related transposons if the entire region of *repC* to *strB* was detected by BLAST in a single contig and a *bla*_TEM_ variant (predominately *bla*_TEM-198_) was also detected in the genome. (Note that it is not possible for the complete transposon sequence to be assembled from short reads, as *bla*_TEM_ is separated from the rest of the transposon by repeat copies of IS*26* which cause a break in the assembly graph). A representative strain matching this pattern (401596) was re-assembled with Unicycler^69^ and the connectivity of Tn*6029* genes in the resulting assembly graphs were visualised using Bandage (v0.8.1)^73^.

The distributions of class I integrons with different cassette regions were explored by extracting the DNA sequences spanning from *int1* to *sul1* genes using BLAST and MUMmer (v3.23)^74^. Different *dfrA* alleles were identified within the resulting sequences via BLAST searches of the ARG-ANNOT database^55^. Representatives of each distinct class I integron sequence (defined by the *dfrA* gene carried) were re-assembled with Unicycler^69^ and submitted to the Repository of Antibiotic-resistance Cassettes (RAC) website^75^ for detailed annotation.

### Antimicrobial usage data and correlation with resistance

Data on the use of antimicrobials at each of the seven GEMS sites were collected as part of the original GEMS protocol^1,26^. These data included details of the antimicrobials prescribed to all cases presenting with watery diarrhoea or dysentery at the study clinics and were documented by a member of the GEMS clinical team^26^. Two of the recorded drugs were excluded from the current analysis: pivmecillinam, because it was not used at any discernible level, and metronidazole, which is active against obligate anaerobic protozoa and bacteria only^76^ and therefore does not pertain to *E. coli*, which is intrinsically resistant to this agent. The frequency of prescriptions for each drug at each site was visualized in R, using the ggplot2 package (v2.2.1)^33^.

The relationship between frequencies of ciprofloxacin and trimethoprim usage and associated genetic determinants was investigated via linear regression modelling in R using the *lm* function. For ciprofloxacin, the genetic determinants were either one or more quinolone resistance-associated point mutations in *gyrA* (point mutations in *parC* only occurred when *gyrA* mutations were also present), or the presence of the plasmid-borne genes *qepA* or *qnrS*. Genetic evidence of trimethoprim/sulphamethoxazole resistance required the combination of at least one *dfrA* gene together with *sul1* or *sul2*. The data were visualized in R using the ggplot2 package^33^.

### Data availability

Accession numbers for the short-read data and associated metadata are listed in Supplementary Table 1. The phenotypic resistance data are provided in Supplementary Tables 3 and 7 and the genotypic resistance profiles are shown in Supplementary Table 4.

## Supplementary information

Supplementary Information Supplementary Note, Supplementary Figures 1–3, Supplementary Tables 2, 5 and 6.

Reporting Summary

Supplementary Table 1 Details of 185 isolates of atypical enteropathogenic *E. coli* analysed in this study.

Supplementary Table 3 Antimicrobial susceptibility profiles of all isolates, inferred from VITEK and agar plate dilution methods.

Supplementary Table 4 Presence/absence of point mutations, acquired AMR genes and plasmid replicon genes in all isolates.

Supplementary Table 7 MIC data from VITEK2 for 185 isolates of atypical enteropathogenic *E. coli* (aEPEC).
