# Supplementary material for: Dynamics of antimicrobial resistance in intestinal Escherichia coli from children in community settings in South Asia and sub-Saharan Africa
Source: Nat Microbiol. 2018 Aug 20;3(9):1063–73. doi: 10.1038/s41564-018-0217-4 (PMC6787116; doi:10.1038/s41564-018-0217-4)
Supplement: Supplementary file 1 — Supplementary Note, Supplementary Figures 1–3, Supplementary Tables 2, 5 and 6. [file 41564_2018_217_MOESM1_ESM.pdf]

nature microbiology

SUPPLEMENTARY INFORMATION
https://doi.org/10.1038/s41564-018-0217-4

In the format provided by the authors and unedited.

Dynamics of antimicrobial resistance in intestinal *Escherichia coli* from children in community settings in South Asia and sub-Saharan Africa

Danielle J. Ingle[1,2,6,7], Myron M. Levine[3], Karen L. Kotloff[3], Kathryn E. Holt[2,4,8] and Roy M. Robins-Browne[5,8*]

[1]Department of Microbiology and Immunology, Peter Doherty Institute for Infection and Immunity, The University of Melbourne, Melbourne, Victoria, Australia. [2]Department of Biochemistry and Molecular Biology, Bio21 Molecular Science and Biotechnology Institute, The University of Melbourne, Melbourne, Victoria, Australia. [3]Departments of Pediatrics and Medicine, Center for Vaccine Development, University of Maryland School of Medicine, Baltimore, MD, USA. [4]London School of Hygiene and Tropical Medicine, London, UK. [5]Murdoch Children's Research Institute, Royal Children's Hospital, Parkville, Victoria, Australia. [6]Present address: National Centre for Epidemiology and Population Health, Research School of Population Health, The Australian National University, Canberra, Australian Capital Territory, Australia. [7]Present address: Microbiological Diagnostic Unit Public Health Laboratory, Department of Microbiology and Immunology, Peter Doherty Institute for Infection and Immunity, The University of Melbourne, Melbourne, Victoria, Australia. [8]These authors contributed equally to this work: Kathryn E. Holt, Roy M. Robins-Browne. *e-mail: r.browne@unimelb.edu.au

Dynamics of antimicrobial resistance in intestinal *Escherichia coli* from children in community settings in South Asia and sub-Saharan Africa

Danielle J. Ingle, Myron M. Levine, Karen L. Kotloff,

Kathryn E. Holt and Roy M. Robins-Browne

Supplementary Note

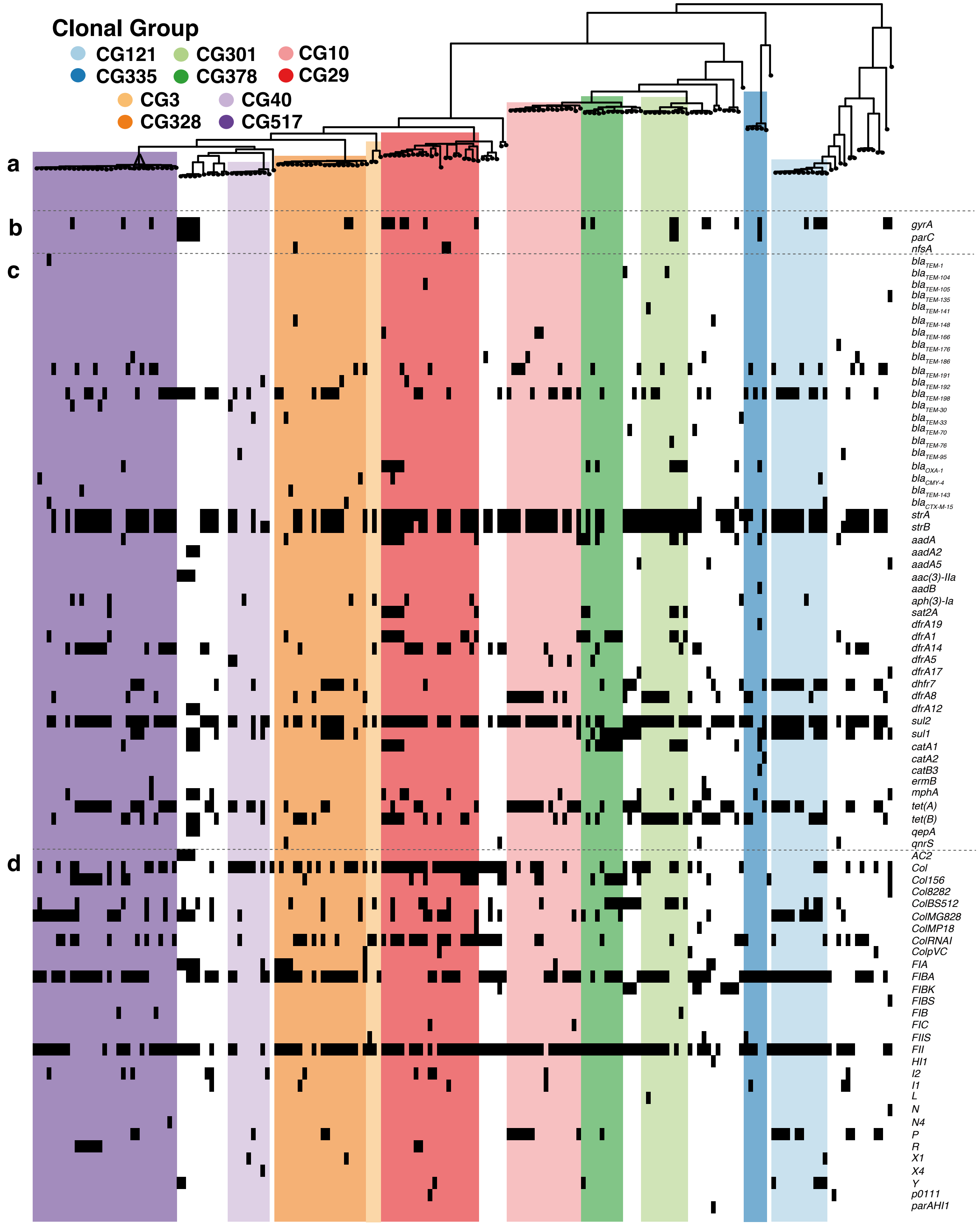
Clonal Group
CG121
CG301
CG10
CG335
CG378
CG29
CG3
CG40
CG328
CG517
a
b
c
d
gyrA
parC
nfsA
blaTEM-1
blaTEM-104
blaTEM-105
blaTEM-135
blaTEM-141
blaTEM-148
blaTEM-166
blaTEM-176
blaTEM-186
blaTEM-191
blaTEM-192
blaTEM-198
blaTEM-30
blaTEM-33
blaTEM-70
blaTEM-76
blaTEM-95
blaOXA-1
blaCMY-4
blaTEM-143
blaCTX-M-15
strA
strB
aadA
aadA2
aadA5
aac(3)-IIa
aadB
aph(3)-Ia
sat2A
dfrA19
dfrA1
dfrA14
dfrA5
dfrA17
dhfr7
dfrA8
dfrA12
sul2
sul1
catA1
catA2
catB3
ermB
mphA
tet(A)
tet(B)
qepA
qnrS
AC2
Col
Col156
Col8282
ColBS512
ColMG828
ColMP18
ColRNAI
ColpVC
FIA
FIBA
FIBK
FIBS
FIB
FIC
FIIS
FII
HI1
I2
I1
L
N
N4
P
R
X1
X4
Y
p0111
parAHI1

2.

Supplementary Figure 1. Presence of common acquired antimicrobial resistance (AMR) genes and plasmid replicon genes in the GEMS aEPEC population. (a) The population structure of 185 EPEC isolates is shown at the top of the figure (sub-setted from core chromosomal phylogeny, see Methods). The ten common aEPEC clonal groups (CG) are depicted by different colours, as per the inset colour key. The presence of AMR genetic determinants are plotted as a binary heatmap with black indicating the presence within each bacterial genome of: (b) point mutations associated with AMR, (c) genes associated with AMR acquired by horizontal gene transfer, and (d) plasmid replicon genes.

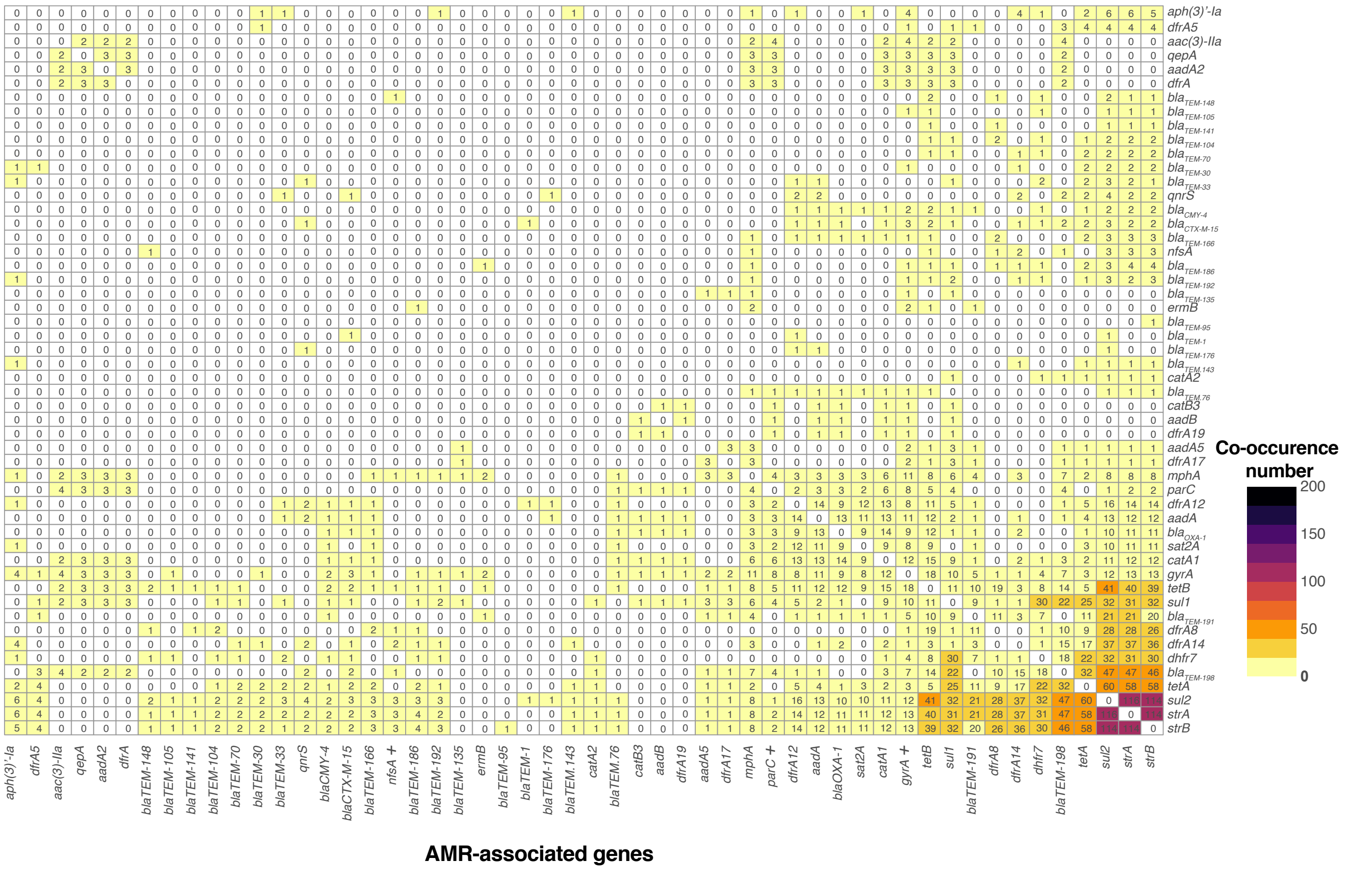

Supplementary Figure 2. Co-occurrence matrix of acquired antimicrobial resistance genes. Number of aEPEC genomes containing different combinations of resistance-encoding genes. These data were used to develop the co-occurrence networks in Figure 3.

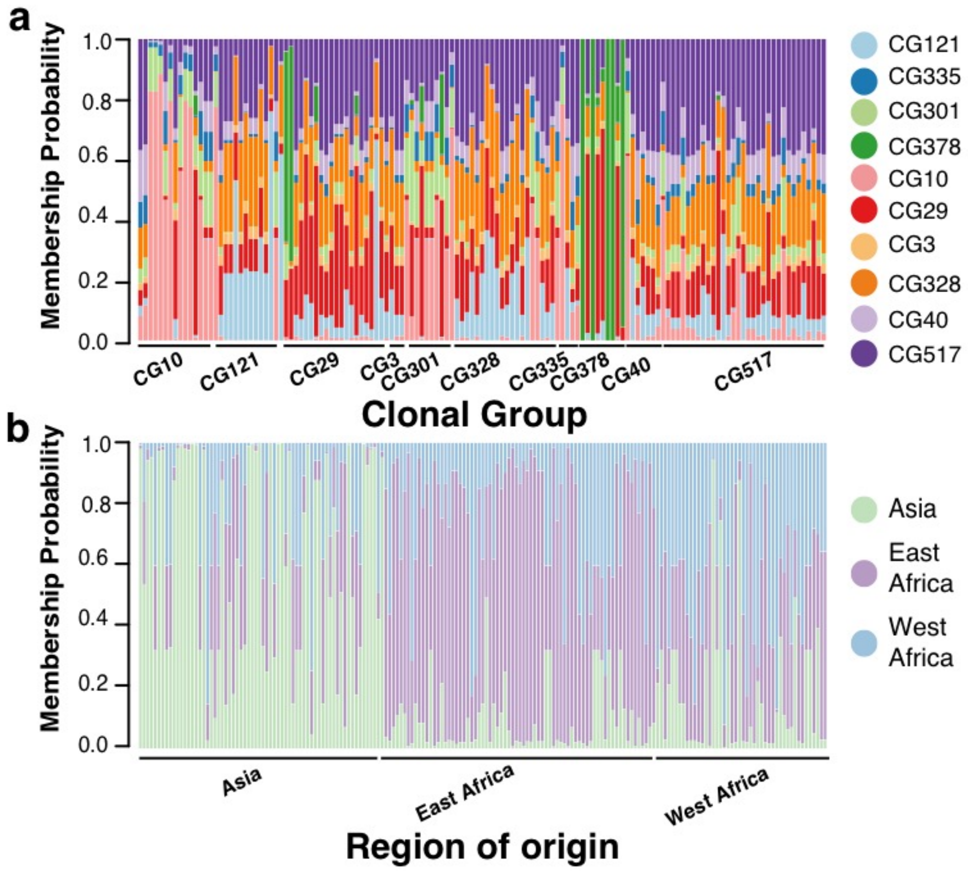

Supplementary Figure 3. Discriminant analysis of principal components (DAPC) group membership probabilities, indicating discrimination of isolates into groups on the basis of the AMR gene content matrix. Groupings subjected to DAPC analysis were (a) clonal groups (CG) (n = 137), and (b) region of origin (n = 185). Each vertical line represents a single isolate, and the colours indicate the probability of assignment to each of the possible groups based on the isolate's AMR gene profile.

Supplementary Tables

The following tables are included with this file:

Supplementary Table 2: MIC breakpoint values used to determine phenotypic resistance

Supplementary Table 5: Predictive power of AMR-associated genotypes for AMR phenotypes for 185 aEPEC

Supplementary Table 6: Frequency of antimicrobial resistance by drug class, stratified by confirmed diseases status (n= 182)

The tables listed below are in separate xlsx files available for download:

Supplementary Table 1. Details of 185 isolates of atypical enteropathogenic *E. coli* analysed in this study.

Supplementary Table 3. Antimicrobial susceptibility profiles of all isolates, inferred from VITEK and agar plate dilution methods.

Supplementary Table 4. Presence/absence of point mutations, acquired antimicrobial resistance (AMR) genes and plasmid replicon genes in all isolates.

Supplementary Table 7. MIC data from VITEK2 for 185 isolates of atypical enteropathogenic *E. coli*
(aEPEC).

Supplementary Table 2: MIC breakpoint values used to determine phenotypic resistance

Antimicrobial	Method	MIC breakpoint	Susceptible ≤ (µg/mL)	Intermediate (µg/mL)	Resistant > (µg/mL)
**Beta-Lactam**					
Ampicillin	VITEK2	EUCAST	8	NA*	8
Ceftazidime	VITEK2	EUCAST	1	4	4
Ceftriaxone	VITEK2	EUCAST	1	2	2
Cefepime	VITEK2	EUCAST	1	4	4
Meropenem	VITEK2	EUCAST	2	8	8
**Aminoglycoside**					
Streptomycin ^	Agar-dilution (16 µg/mL)	Neither	8	NA	≥16
Gentamicin	VITEK2	EUCAST	2	4	4
Tobramycin	VITEK2	EUCAST	2	4	4
Amikacin	VITEK2	EUCAST	8	16	16
**Folate Pathway Inhibitors**					
Trimethoprim	VITEK2	EUCAST	2	4	4
Trimethoprim/ sulphamethoxazole ^^	VITEK2	EUCAST	2	4	4
**Nitrofurantoin**					
Nitrofurantoin	VITEK2	EUCAST	64	NA	64
**Chloramphenicol**					
Chloramphenicol	Agar-dilution (16 µg/mL)	EUCAST	8	NA	8
**Macrolides**					
Azithromycin^	Agar-dilution (16 µg/mL)	Neither	8	NA	≥16
**Tetracyclines**					
Tetracycline #	Agar-dilution (8 µg/mL)	CLSI	4	8	16
**Fluoroquinolones**					
Ciprofloxacin	VITEK2	EUCAST	0.5	1	1
Norfloxacin	VITEK2	EUCAST	0.5	1	1

* NA, not applicable

^ The breakpoint for *E. coli* is uncertain and is not provided by EUCAST or CLSI. A resistance breakpoint of MIC 16 µg/mL was used in keeping with that used for *Salmonella enterica*.

^^ Ratio of 1:19 used for trimethoprim:sulphamethoxazole

CLSI cut-off used as no MIC breakpoint given under EUCAST. Intermediate and resistant phenotyp

Supplementary Table 5: Predictive power of AMR-associated genotypes for AMR phenotypes for 185 aEPEC

Antimicrobial	Sensitivity (95% CI)	Specificity (95% CI)	PPV (95% CI)	NPV (95% CI)
**Beta-lactam**				
Ampicillin	0.93 (0.86 - 0.97)	0.80 (0.68 - 0.89)	0.90 (0.83 - 0.94)	0.85 (0.73 - 0.93)
Cefepime	1 (0.29 - 1)	0.98 (0.94 - 0.99)	0.43 (0.1 - 0.82)	1 (0.98 - 1)
Ceftazidime	1 (0.54 - 1)	0.97 (0.94 - 0.99)	0.55 (0.23 - 0.83)	1 (0.98 - 1)
Ceftriaxone	1 (0.54 - 1)	0.97 (0.94 - 0.99)	0.55 (0.23 - 0.83)	1 (0.98 - 1)
Meropenem	NA	1 (0.98 - 1)	NA	1 (0.98 - 1)
**Aminoglycoside**				
Streptomycin	0.95 (0.88 - 0.99)	0.53 (0.43 - 0.63)	0.61 (0.52 - 0.69)	0.93 (0.84 - 0.98)
Gentamicin	1 (0.48 - 1)	1 (0.98 - 1)	1 (0.48 - 1)	1 (0.98 - 1)
Tobramycin	1 (0.48 - 1)	1 (0.98 - 1)	1 (0.48 - 1)	1 (0.98 - 1)
Amikacin	NA	1 (0.98 - 1)	NA	1 (0.98 - 1)
**Folate Pathway Inhibitor**				
Trimethoprim	0.97 (0.92 - 0.99)	0.82 (0.7 - 0.91)	0.92 (0.85 - 0.96)	0.93 (0.82 - 0.98)
Trimethoprim/Sulphonamide	0.97 (0.92 - 0.99)	0.84 (0.73 - 0.92)	0.92 (0.86 - 0.96)	0.93 (0.83 - 0.98)
**Nitrofurantoin**				
Nitrofurantoin	NA	0.98 (0.95 - 1)	0 (0 - 0.71)	1 (0.98 - 1)
**Chloramphenicol**				
Chloraphenicol	0.9 (0.7 - 0.99)	0.98 (0.94 - 0.99)	0.83 (0.61 - 0.95)	0.99 (0.96 - 1)
**Macrolides**				
Azithromycin	0.85 (0.55 - 0.98)	0.97 (0.93 - 0.99)	0.65 (0.38 - 0.86)	0.99 (0.96 - 1)
**Tetracyclines**				
Tetracycline	0.96 (0.9 - 0.99)	0.9 (0.81 - 0.96)	0.93 (0.86 - 0.97)	0.95 (0.87 - 0.99)
**Fluoroquinolone**				
Ciprofloxacin	1 (0.63 - 1)	0.98 (0.94 - 0.99)	0.67 (0.35 - 0.9)	1 (0.98 - 1)
Norfloxacin	0.97 (0.83 - 1)	0.97 (0.93 - 0.99)	0.86 (0.7 - 0.95)	0.99 (0.96 - 1)

Point estimates for Sensitivity, Specificity, Positive Predictive Value (PPV) and Negative Predictive Value (NPV) with 95% confidence intervals

NA, not applicable

Supplementary Table 6: Frequency of antimicrobial resistance by drug class, stratified by confirmed diseases status (n= 182)

Drug Class	Phenotypic resistance				Resistance predicted from genotype			
	% Cases (n=94)	% Controls (n=88)	*P*-value (Fisher's exact test)	Odds Ratio [95% CI]	% Cases (n=94)	% Controls (n=88)	*P* value (Fisher's exact test)	Odds Ratio [95% CI]
**Ampillicin**	71.3	60.2	0.1	0.6 [0.3-1.2]	73.4	62.5	0.2	0.6 [0.3-1.2]
**3rd-4th gen. cephalosporins**	3.2	2.3	1	0.7 [0.1-6.3]	6.4	4.5	0.7	0.7 [0.1-3.1]
**Meropenem**	0.0	0.0	-	-	0.0	0.0	-	-
**Aminogylcoside**	46.8	45.5	0.9	0.9 [0.5-1.8]	72.3	76.1	0.6	1.2 [0.6-2.5]
**Amikacin**	0.0	0.0	-	-	0.0	0.0	-	-
**Trimethoprim**	68.1	64.8	0.6	0.9 [0.4-1.7]	72.3	68.2	0.6	0.8 [0.4-1.6]
**Trimethoprim/ Sulphonamide**	67.0	63.6	0.6	0.9 [0.4-1.7]	72.3	71.6	1	1 [0.5-1.9]
**Nitrofurantoin**	-	-	-	-	1.1	2.3	0.6	2.2 [0.1-128.8]
**Chloramphenicol**	16.0	6.8	0.1	0.4 [0.1-1.1]	17.0	8.0	0.1	0.4 [0.1-1.2]
**Macrolide**	8.5	5.7	0.6	0.6 [0.2-2.4]	8.5	10.2	0.8	1.2 [0.4-3.8]
**Tetracyclines**	52.1	60.2	0.3	1.4 [0.7-2.6]	55.3	61.4	0.5	1.3 [0.7-2.4]
**Fluoroquinolones**	19.1	13.6	0.4	0.7 [0.3-1.6]	19.1	18.2	1	0.9 [0.4-2.1]
