# Supplementary material for: Dynamics of antimicrobial resistance in intestinal Escherichia coli from children in community settings in South Asia and sub-Saharan Africa
Source: Nat Microbiol. 2018 Aug 20;3(9):1063–73. doi: 10.1038/s41564-018-0217-4 (PMC6787116; doi:10.1038/s41564-018-0217-4)
Supplement: Supplementary file 2 — Reporting Summary [file 41564_2018_217_MOESM2_ESM.pdf]

nature research

Corresponding author(s): *Double-blind peer review submissions: write DBPR and your manuscript number here instead of author names.*

Reporting Summary

Nature Research wishes to improve the reproducibility of the work that we publish. This form provides structure for consistency and transparency in reporting. For further information on Nature Research policies, see Authors & Referees and the Editorial Policy Checklist.

Statistical parameters

When statistical analyses are reported, confirm that the following items are present in the relevant location (e.g. figure legend, table legend, main text, or Methods section).

n/a	Confirmed	
☐	☒	The exact sample size (*n*) for each experimental group/condition, given as a discrete number and unit of measurement
☐	☒	An indication of whether measurements were taken from distinct samples or whether the same sample was measured repeatedly
☐	☒	The statistical test(s) used AND whether they are one- or two-sided<br>*Only common tests should be described solely by name; describe more complex techniques in the Methods section.*
☐	☒	A description of all covariates tested
☒	☐	A description of any assumptions or corrections, such as tests of normality and adjustment for multiple comparisons
☐	☒	A full description of the statistics including central tendency (e.g. means) or other basic estimates (e.g. regression coefficient) AND variation (e.g. standard deviation) or associated estimates of uncertainty (e.g. confidence intervals)
☐	☒	For null hypothesis testing, the test statistic (e.g. *F*, *t*, *r*) with confidence intervals, effect sizes, degrees of freedom and *P* value noted<br>*Give P values as exact values whenever suitable.*
☒	☐	For Bayesian analysis, information on the choice of priors and Markov chain Monte Carlo settings
☒	☐	For hierarchical and complex designs, identification of the appropriate level for tests and full reporting of outcomes
☒	☐	Estimates of effect sizes (e.g. Cohen's *d*, Pearson's *r*), indicating how they were calculated
☐	☒	Clearly defined error bars<br>*State explicitly what error bars represent (e.g. SD, SE, CI)*

Our web collection on statistics for biologists may be useful.

Software and code

Policy information about availability of computer code

Data collection	Not applicable
Data analysis	adegenet package (v2.1.0); pheatmap package (v1.0.8); igraph package (v1.1.2); ape (v5.1); geiger (v2.0.6); genoplotR (v0.8.7); epiR package (v0.9-93); Unicycler (v0.2.0); Prokka (v1.12); Bandage (v0.8.1); MUMmer (v3.23); Artemis Comparison Tool; BLAST (v2.3.30).

For manuscripts utilizing custom algorithms or software that are central to the research but not yet described in published literature, software must be made available to editors/reviewers upon request. We strongly encourage code deposition in a community repository (e.g. GitHub). See the Nature Research guidelines for submitting code & software for further information.

Data

Policy information about availability of data

All manuscripts must include a data availability statement. This statement should provide the following information, where applicable:

- Accession codes, unique identifiers, or web links for publicly available datasets
- A list of figures that have associated raw data
- A description of any restrictions on data availability

Accession numbers for the short-read data and associated metadata are listed in Supplementary Table 1. The phenotypic resistance data are provided in Supplementary Tables 3 and 7 and the genotypic resistance profiles are shown in Supplementary Table 4.

Field-specific reporting

Please select the best fit for your research. If you are not sure, read the appropriate sections before making your selection.

☒ Life sciences ☐ Behavioural & social sciences ☐ Ecological, evolutionary & environmental sciences

For a reference copy of the document with all sections, see nature.com/authors/policies/ReportingSummary-flat.pdf

Life sciences study design

All studies must disclose on these points even when the disclosure is negative.

Sample size	No sample size calculation was performed. As described in the methods section, we used all of the bacterial strains that were available from the original GEMS study
Data exclusions	As described in the MS, for some analyses we excluded samples where the case/control status of the isolate was unknown.
Replication	Not applicable
Randomization	Not applicable
Blinding	Resistance phenotypes were determined and recorded without knowledge of the genotype. Antibiotic usage data were examined only after all the phenotypes and genotypes related to AMR had been analysed and recorded. All other data were computer-generated and not adjusted.

Reporting for specific materials, systems and methods

Materials & experimental systems

n/a	Involved in the study	
☐	☒	Unique biological materials
☒	☐	Antibodies
☒	☐	Eukaryotic cell lines
☒	☐	Palaeontology
☒	☐	Animals and other organisms
☐	☒	Human research participants

Methods

n/a	Involved in the study	
☒	☐	ChIP-seq
☒	☐	Flow cytometry
☒	☐	MRI-based neuroimaging

Unique biological materials

Policy information about availability of materials

Obtaining unique materials	All unique materials are available from the authors.

Human research participants

Policy information about studies involving human research participants

Population characteristics	The bacteria used in this study were of human origin. The way patients and controls were selected and the way samples were

Population characteristics	collected have been described in Kotloff et al. Lancet 382, 209–222 (2013); Panchalingam et al. Clin Infect Dis 55, S294–S302 (2012), and Ingle et al. Nat Microbiol 1, 15010 (2016).
Recruitment	See above
